# Familiarity with humans affect dogs’ tendencies to follow human majority groups

**DOI:** 10.1038/s41598-020-64058-5

**Published:** 2020-04-28

**Authors:** Miho Nagasawa, Kazutaka Mogi, Hisashi Ohtsuki, Takefumi Kikusui

**Affiliations:** 1 Azabu University, Department of Animal Science and Biotechnology, Sagamihara, 252-5201 Japan; 2 Department of Evolutionary Studies of Biosystems, School of Advanced Sciences, SOKENDAI (The Graduate University for Advanced Studies), Hayama, 240-0193 Japan

**Keywords:** Psychology, Zoology

## Abstract

Recently, copying others’ behaviour has attracted attention among researchers. It aids individuals in reducing uncertainty about the knowledge of the environment and helps them in acquiring an adaptive behaviour at a lower cost than by learning it by themselves. Among the copying strategies, conformity, which is the copying of behavioural decisions presented by the majority, has been well studied and reported in many animals, including humans. The previous study showed that dogs did not conform to their multiple conspecific individuals; however, dogs have evolved to increase their adaptability while living with humans, and it is plausible that dogs have selected appropriate behaviour according to the behaviour of humans. Therefore, we investigated which factors influenced the choice of dogs in a situation where they have to choose one of two numerically unbalanced human groups. The results showed that the dogs followed the human majority group under certain conditions, depending on the familiarity with the human demonstrators. These results are important in considering the significance of groups for dogs and the factors of group formation, and will also provide a clue as to how dogs have penetrated into human society.

## Introduction

Dogs (*Canis familiaris*) have coexisted with humans for more than 30,000 years and are woven into human society as partners to humans. Interactions between dogs and humans affect both species’ endocrine systems and produce a positive loop of bonding via oxytocin^1^. This oxytocinergic bonding is commonly found in strong intraspecies connections, such as those between mother–infant and mating partners^2^, and it contributes to the survival of themselves and their offspring. Dogs are suggested to have had acquired human-like communication skills as a by-product of the mutation of the stress response endocrine system during the domestication process^3^, and as a result, humans and dogs may have become able to coexist by applying this bonding system to each other beyond species. Dogs have high sensitivity to human gaze direction and attention status^4–8^ and change their behaviour depending on humans. They often naturally behave similarly to humans, and prefer humans who synchronise with them^9^. They can also copy human actions at various levels, such as mimicry, automatic imitation, behavioural synchronisation, selective imitation, and over-imitation, which may also indicate a high sensitivity of dogs to human behaviour^10–16^.

In decision-making, copying the behaviours of others is an effective way to reduce uncertainty about the environmental knowledge, and can help individuals to acquire an adaptive behaviour at a lower cost than by learning it by themselves. Among the copying strategies, conformity, that is copying the behavioural decision presented by the majority, has been well studied in many animals^17,18^. Since conformity was first reported in humans, some studies were conducted on primates, such as comparisons among human infants, chimpanzees, and orangutans, to investigate its evolutionary root^19,20^. Other species in different phylogenetic branches from humans have also been examined from the perspective of the convergent evolution of conformity^21–23^. As van Leeuwen and Haun^24^ defined, conformity is the tendency to forgo personal information by adopting the cultural variant that is used by the majority, so it is controversial whether some non-human studies match the definition of conformity or not^18^. Nevertheless, the behavioural adaptation of copying the majority choice could exist in animals living in groups under certain conditions.

For example, wolves form packs based on kinship and show sophisticated cooperation in hunting and breeding. Usually, the breeding parents, which are the oldest and most experienced in the pack, are more likely to lead the pack during travel or in the pursuit of pray. Bigger hunting groups have a higher hunting success rate; especially in the presence of scavengers of other species, the larger the group of wolves is, the more advantageous it is for hunting. However, too large a group appears to reduce hunting success because of increased free-riders^25,26^. The success of hunting in wolves might depend on the perception of movement of other members and the numerical balance in the pack; therefore, there is a strong possibility that wolves show conformity to their conspecifics. Germar *et al*.^27^, however, examined conformity in dogs, which have a common ancestor with wolves, using the same method as Haun *et al*.^19^, and found that dogs do not copy behaviour presented by multiple conspecific individuals, but stick to what they have learned. Previous studies have also shown that domesticated dogs do not have cooperative behaviour with conspecifics either in a natural environment or under experiment, but wolves do^28,29^. Interestingly, dogs can cooperate with human partners in a string-pulling task as well as wolves can; however, wolves were more likely to initiate movement leading the interaction with humans, whereas dogs were more likely to wait for the human to initiate actions and then follow^30^. Dogs were also reported to refer more to information from their owner than their conspecifics living in the same household^31^. Therefore, dogs could be considered to have decreased their requirements to refer to or follow conspecifics during the process of domestication. It may be reasonable to think that it is advantageous for dogs to obey humans rather than conspecifics. Actually, free-living dogs in urban areas are mainly solitary or in pairs, which seems to be caused by lower motivation to contact with conspecifics due to greater bonds with owners and other humans compared to dogs in rural areas^32^.

For numerical competence in dogs that is the premise of choice based on group size, West and Young^33^ investigated quantitative representations in dogs using the violation of expectancy paradigm, and found positive results. Ward and Smuts^34^ and Barker^35^ used the two-box spontaneous choice paradigm and found that dogs could discriminate between two small quantities in agreement with Weber’s Law, although there are numerical limitations. Dogs’ numerical competence has been examined in food choice tests, but not in human choice tests. However, when two cups were presented to dogs and were pointed at by one and two informants respectively, dogs chose the cup pointed at by the two informants, even if it was a non-baited cup; therefore, it is considered that dogs can count the number of humans and try to obtain information from multiple humans^36^. From these results, dogs are expected to be able to choose human groups based on the number of group members.

From the above, we hypothesised that i) in a situation where dogs have to choose one of two human groups, dogs would tend to choose the larger sized group more when the difference in the number of members in the two groups was larger in agreement with Weber’s Law^34,35^. On the other hand, short-term contact with humans affect dogs’ behaviour towards humans, so dogs are more likely to follow pre-contacted people than unfamiliar people^37^. Therefore, ii) if the experience of contact with humans is worth more than being in the majority, dogs would choose pre-contacted people even if the people belong to minority groups, instead of the majority in which all humans are unfamiliar. In the current study, we presented dogs with two groups with different numbers of humans and examined which group the dogs chose. Dogs were allowed to observe the human demonstrators, who were all unfamiliar to the dogs, separated into two groups with different numbers; the majority and the minority, which were divided by different ratios, such as 5:1, 4:2, and 3:2. The dogs were then released and the group that was chosen was recorded. The same experimental procedure was performed in which members of the minority human group were familiar to the dogs in order to examine whether the dogs’ familiarity toward demonstrators influenced their choice. We conducted these procedures with both house dogs and shelter dogs because the dogs’ living environment can affect their behaviour^38,39^, especially regarding their relationship with humans^40^.

## Results

**Figure 1:**
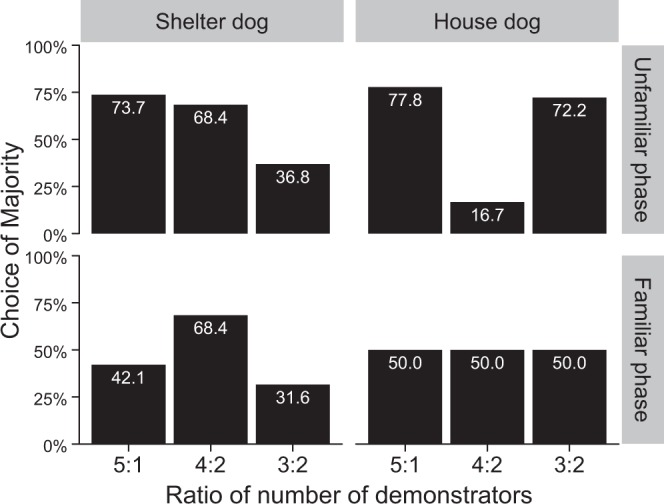
Results of test session. The bars show the percentage of dogs that chose the majority group.

Figure 1 shows the percentage of majority choice for different conditions. In the unfamiliar phase, more than half shelter dogs chose the majority in the 5:1 (73.7%) and 4:2 ratios (68.4%), however, the number of shelter dogs that chose the majority decreased in the 3:2 ratio (36.8%). On the other hand, house dogs showed high majority selection rates in the 5:1 (77.8%) and 3:2 ratios (72.2%) but not for 4:2 ratio (16.7%). In the familiar phase, except for shelter dogs in 4:2 ratio (68.4%), the majority selection rate was at most 50% (shelter dogs 5:1 ratio: 42.1%, 3:2 ratio: 31.6%, house dogs in all ratios showed 50%).

We performed a generalised linear mixed model (GLMM) analysis (multivariate logistic regression with mixed effects) to examine which factors influenced the choice of dogs. To predict the choice of dogs, we considered the following variables as fixed effects: dog’s sex, age, familiarity with demonstrators (unfamiliar/familiar phase), history (house dog/shelter dog), and the ratio of demonstrators (5:1/4:2/3:2). The order of experiments (unfamiliar or familiar phase first) was also included as a control variable. We included the interactions between familiarity and ratio of demonstrators, and between history and ratio of demonstrators in the model, because we hypothesised that how dogs interact with humans/demonstrators could affect their behaviour. In addition, we also examined all the other first-order interactions between familiarity, history, ratio, and other variables. The dog’s identity was included as a random effect.

As a result of our GLMM analysis, we found no significant main effects in any of the variables. However, significant interactions were found between familiarity and ratio (*p* = 0.016) and between history and ratio (*p* < 0.001). These main effects and interactions are summarised in Table 1. The tests of simple main effect between familiarity and ratio showed that the dogs in the unfamiliar phase chose the majority significantly more than the dog in the familiar phase when the ratio of demonstrators was 5:1 ratio (*p* = 0.037). The significant simple main effects between history and ratio are as follows. Shelter dogs chose the majority more than house dogs at the 4:2 ratio (*p* = 0.048). House dogs chose the majority more at the 5:1 ratio than at the 4:2 ratio (*p* = 0.036), and they also chose the majority more at the 3:2 ratio than at the 4:2 ratio (*p* = 0.022) (Table 2).

**Table 1:** Summary of GLMM analysis.

Variables	Chi-squared	df	p-value
**GLMM by glmer:** **formula = cbind(Choice, 1 − Choice) ~ (Sex + Age + Order + Familiarity + History + Ratio)**^**2**^ **+ (1|Name)**			
Sex	0.9468	1	0.3305
Age	1.6177	1	0.2034
Order	0.4856	1	0.4859
Familiarity	1.6806	1	0.1949
History	0.0758	1	0.7831
Ratio	2.0619	2	0.3567
Sex: Age	0.1816	1	0.6700
Sex: Order	0.0339	1	0.8539
Sex: Familiarity	0.4433	1	0.5055
Sex: History	1.5343	1	0.2155
Sex: Ratio	1.6105	2	0.4470
Age: Order	0.0819	1	0.7747
Age: Familiarity	0.2961	1	0.5863
Age: History	0.2597	1	0.6103
Age: Ratio	1.0843	2	0.5815
Order: Familiarity	2.2825	1	0.1308
Order: History	1.7990	1	0.1798
Order: Ratio	0.5108	2	0.7746
Familiarity: History	0.0531	1	0.8177
Familiarity: Ratio	8.2999	2	*0.0158
History: Ratio	16.4715	2	***0.0003

Results of Type-II Wald chi-square test is shown.

**Table 2:** Post-hoc tests for simple main effects of GLMM analysis.

condition	contrast tested	coefficient	Chi-squared	(adjusted) p-value	note
**Post-hoc tests for Familiarity: Ratio**					
Ratio = 5:1	Familiar - Unfamiliar	−2.2015	4.3574	*0.0368	
Ratio = 4:2	Familiar - Unfamiliar	−0.0540	0.0028	0.9579	
Ratio = 3:2	Familiar - Unfamiliar	−1.5075	2.1293	0.1445	
Familiarity = Unfamiliar	4:2–5:1	−0.7426	0.5705	1	(a)
	3:2–5:1	−0.8306	0.7352	1	
	3:2–4.2	−0.0881	0.0084	1	
Familiarity = Familiar	4:2–5:1	1.4048	2.3307	0.3805	(a)
	3:2–5:1	−0.1367	0.0237	1	
	3:2–4.2	−1.5415	2.7655	0.2890	
**Post-hoc tests for History: Ratio**					
Ratio = 5:1	House - Shelter	0.0642	0.0043	0.9478	
Ratio = 4:2	House - Shelter	−1.9535	3.9215	*0.0477	
Ratio = 3:2	House - Shelter	1.1104	1.3647	0.2427	
History = Shelter	4:2–5:1	−0.7426	0.5705	1	(a)
	3:2–5:1	−0.8306	0.7352	1	
	3:2–4.2	−0.0881	0.0084	1	
History = House	4:2–5:1	−2.7603	6.3009	*0.0362	(a)
	3:2–5:1	0.2155	0.0397	1	
	3:2–4.2	2.9758	7.1826	*0.0221	

Results of Wald-test with chi-squared statistic, two-sided.

(a) p-values were Bonferroni-adjusted for multiple comparisons.

## Discussion

In recent years, dogs’ human-like communication skills have attracted attention. It has been found that dogs are highly sensitive to and can refer to human behaviour and emotion, and the degree of dogs’ experience with humans influences these abilities^41^. In the current study, in view of the specific relationship between dogs and humans, we investigated whether dogs have an orientation toward human majority, which is generally found in intraspecific groups. We hypothesised that dogs would choose the larger group (majority) when the size of the presented groups was greatly biased; however, manipulation of familiarity with the group members would diminish that tendency. We found the significant interaction between familiarity with demonstrators and the ratio of demonstrators, and post-hoc tests for simple main effects showed that the proportion of majority choice at the ratio of 5:1 was lower in the familiar phase (45.95%, 17/37 dogs) than that in the unfamiliar phase (75.68%, 28/37 dogs). These results suggested that the familiarity with demonstrators can affect majority choice in dogs, as hypothesised. However, we did not find any linear trend that was dependent on the ratio of demonstrators according to Weber’s law although shelter dogs showed this trend in the unfamiliar phase.

In the familiar phase, the probability that dogs chose the majority group was not above 50%, except for shelter dogs in the trial with a 4:2 ratio. In Marshall-Pescini’s study^42^, when presented with two plates with different quantity of food, nearly 80% of dogs chose the plate with more food for more than two trials out of three; however, a half of the dogs chose the plates with a smaller quantity for more than two trials out of three when the owners walked towards the smaller quantity plate. In the current experiment, the dogs had learned beforehand in the warm-up session that they received food as long as they followed demonstrators. Moreover, the amount of food in the two containers is the same and the dogs could not see the food in the container at the starting position, so there were no counteractive factors, such as the difference in food quantity in the previous study. Therefore, we expected that most dogs would follow the minority group (familiar demonstrators), both in house dogs and shelter dogs. As a result, we found that becoming acquainted with demonstrators of minority group in advance of the test weakened the likelihood of dogs choosing the majority, as hypothesized; however, it has not always encouraged most dogs to choose the familiar demonstrators. Reduction of dog’s motivation is one of the possibilities that could explain why a half of the dogs did not choose the familiar demonstrators in the familiar phase even though the difference in the bias of group size was small. The experiments were conducted after teaching them that they could receive food as long as they followed humans, no matter which container they chose. This is to ensure that differences in dog’s experiences about food acquisition did not affect their choices during the session, because it is known that dogs have large individual differences depending on their life environment and experience^43,44^. Therefore, the importance for the dogs to choose one group over the other based on the number of human demonstrators may have decreased. It is also possible that there were dogs that did not associate food in the containers with demonstrators. However, these interpretations contradict the fact that 75.7% of dogs chose the majority group at the ratio of 5:1 in the unfamiliar phase which were conducted under the same conditions. Especially, 16 of 20 dogs which chose the minority group in the familiar phase chose the majority in the unfamiliar phase; therefore, these interpretations may not be possible to explain all of the results. The other possibility is that a larger-sized human group is more attractive for a half of dogs than the existence of familiar persons. We did not find any difference in dog’s age, sex, history between the dogs that followed the familiar demonstrators and those which did not. In the current study, the dogs contacted with the familiar demonstrators for only 10 minutes just before the trials. Such quality or quantity of contact with humans may not have been enough for some dogs. While there are individual differences, the degree of relationship with the demonstrators can be an important factor for dogs in their decision-making.

In Zimen’s pilot study^45^, if a group was divided into two unbalanced groups (with the leader in the smaller), wolves had a conflict over whether to follow the leader group or the larger group separated from the leader group. On the other hand, young wolves tended to be basically attracted to strangers (both humans and wolves). For the survival of wolves, the number of members in the group must have been an important factor; however, that may have depended on the balance between the benefits of group size and individual relationships with group members. This may correspond to the result in our experiment that the choice of familiar minority groups was around 50%. In our analysis, younger individuals did not especially choose unfamiliar groups over others because dogs’ ages were found not to have a significant effect; however, as it is a general animal habit to show interest in or search for novel objects, there may have been individuals who dared to choose unfamiliar demonstrators in our study according to individual characteristics. Moreover, Zimen’s wolves’ behaviour was also influenced by the environment. They tended to follow strangers in wide plain areas with good visibility, whereas in the forest, they followed the leader group that included the leader wolf and/or human caregiver. In the current study, trials were conducted in an experimental room where the dogs were able to see the demonstrators clearly, so dogs may have followed the unfamiliar demonstrators in the majority and not the familiar ones. Our results may be altered by changing the experiment setting, such as providing obstacles or widening the experiment area, which would increase the cost of choice for dogs. On the other hand, 5 dogs did not choose the majority in the unfamiliar phase but chose it in the familiar phase in the 5:1 ratio, and some dogs also showed the same results in the other ratios (4:2 ratio: 9 dogs, 3:2 ratio: 6 dogs). These are inconsistent with our hypothesis that familiarity to humans affects dog’s majority choices. However, this result may be a consequence of our small sample size. In this study, only one trial per condition was performed; however, more repetitions of trials for each ratio could have allowed us to evaluate the dog’s majority choice more clearly; we would have been able to evaluate the effect of familiarity with the demonstrators despite the existence of individual differences. A review of our experimental methods and a larger sample size are required in the future.

GLMM also showed a significant interaction between dog’s history and ratio. The proportion of dogs that chose the majority group at the 4:2 ratio was significantly smaller in house dogs compared with shelter dogs. This is mainly because the number of house dogs that chose the majority at the 4:2 ratio in the unfamiliar phase was extremely low (16.7%), while those at other ratios were over 70%. Additionally, the proportion of shelter dogs which chose the majority group at the 4:2 ratio was higher (68.4%) than the other ratios in the familiar phase. In this experiment, the demonstrators, who were different each time, were mostly females, and we set the ratio of gender in both groups to be as even as possible if males were included; therefore, it is unlikely that there was a problem in the presentation of the demonstrators only in the 4:2 ratio trials. We are not aware of other factors that may have biased the dogs’ behaviour, so we were unable to find a reasonable explanation as to why dogs changed the tendency of majority choice in the trial with a 4:2 ratio in both house dogs in the unfamiliar phase and shelter dogs in the familiar phase.

In this study, our results suggested that dogs followed the human majority group under certain conditions, which seem to be related to the degree of relationship of human group members which they established. On the other hand, we did not find any linear trend that was dependent on the ratio of demonstrators according to Weber’s law in dog’s majority choices. We taught the dogs to obtain food in advance, so the behaviour of the dogs observed in this study did not exactly mean copying the majority by social learning (including conformity). However, although we could not find statistical evidence, shelter dogs tend to change the choice of the majority group due to differences in the number of group members, which may be considered as adaptive behaviour acquired during the early stage of domestication. As mentioned above, dogs are a unique species that can form interspecies relationships while forming flexible conspecific groups. Though the future studies are required due to the limitation of our experimental methods and small sample size, the factors influencing dog’s majority choice revealed in this study are important in considering the significance of dogs’ group-formation with humans, and these results will provide a clue as to how dogs have merged into human society by weighing the benefit opportunities of choice of majority group or the relationships with humans.

## Methods

### Participants

This study involved 38 dogs (3 intact males, 15 castrated males, 10 intact females, and 10 spayed females; average age: male 4.89 ± S.E. 0.88 years, female 5.05 ± 0.71 years [ages of some shelter dogs were estimated]; Table S1), two female experimenters, and 12 demonstrators per session. The experiments were conducted from 2013 to 2017. Nineteen dogs were house dogs and the rest were from dog shelters. The house dogs were recruited from dog training classes, veterinary clinics, dog parks, and events for dog owners. Written informed consent was obtained from their owners. The shelter dogs were brought from the public dog shelters and kept in the facility in Azabu University for about six months to socialise and rehome them with new owners. The experiments with shelter dogs were conducted about one month after the dogs were brought to the university. Around the same time that the shelter dogs were in the university, we conducted experiments with almost the same number of house dogs. All dogs were healthy, and were not afraid of new places or unfamiliar persons. We excluded one house dog from the analysis because it was afraid of the demonstrators and could not wait at the starting position in the test session.

### Procedures

Experiments were conducted in experimental areas (5 × 5 m) that were divided by tape in the experimental rooms at Azabu University. The experiment consisted of a warm-up session and a test session. The test session comprised two phases, the unfamiliar phase and the familiar phase. Each phase consisted of three trials. The order of the phases was counterbalanced; half of the dogs were first tested in the unfamiliar phase, and the other half were first tested in the familiar phase, and then the groups were swapped. All demonstrators who participated in the test session in the unfamiliar phase had no previous contact with the dogs, while only two of the demonstrators who participated in the familiar phase interacted with and gave food to the dogs in advance. We recorded dog behaviours using a video camera (HDR-AS200V; Sony Corp., Tokyo, Japan).

#### Warm-up session

**Figure 2:**
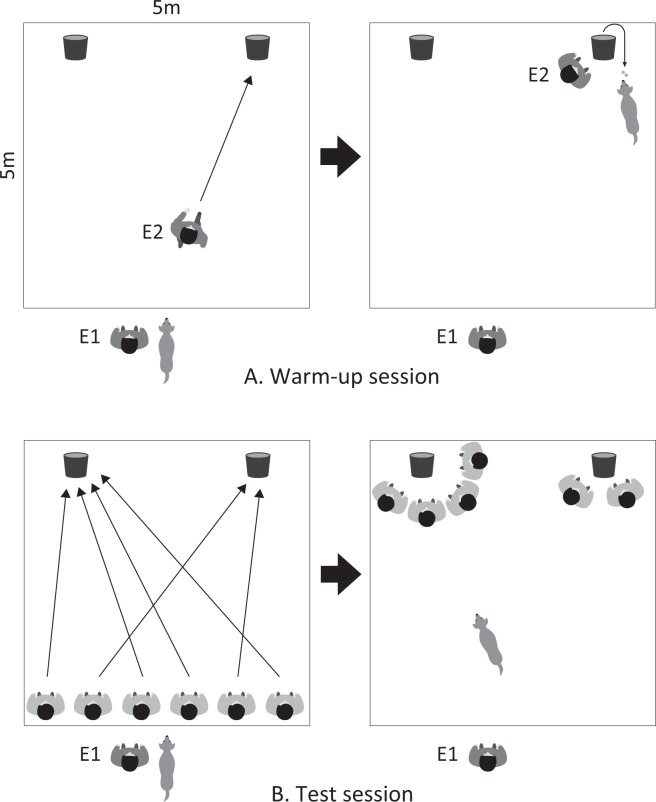
Warm-up and the test session procedures. (**A**) In the warm-up session, Experimenter 2 (E2) walked towards one of the two containers as a counterbalance. The dog obtained food if it followed E2. (**B**) In the test session, the demonstrators were split into two groups, the majority and the minority, and walked towards one of the containers. E1 then released the dog. In the familiar phase, the familiar demonstrators were assigned to the minority group.

The warm-up session aimed to teach the dogs to obtain food by following humans. Dog’s individual training experience has a great influence on dogs’ behaviour, such as problem-solving ability^46^; therefore, in the present study, we taught them in advance that food could be obtained if they followed humans before test session. First, experimenter 1 (E1) kept the dog on a leash at the starting position. Experimenter 2 (E2) set two containers in the centre of the experimental area and put commercial dog treats inside the containers. Then, E1 led the dog toward the containers and allowed it to eat treats from both containers. Next, E1 brought the dog back to the starting position and E2 moved the containers to the two corners on the opposite side of the experimental area to the starting position and put treats inside them. E2 stood at the starting position with her back to the dog and moved towards one of the containers. Shortly after, E1 released the dog and the dog obtained treats from the container when it chose the container that E2 approached (Fig. 2A). These trials were continued until dogs followed E2 and obtained treats four times in succession. E2 approached both containers randomly.

#### Test session

The test session was conducted around 10 min after the dog completed the warm-up session. E2 set two containers in the centre of the experimental area and put treats inside the containers while the dog watched from the starting position. E2 led the dog towards the containers and allowed it to eat treats from both containers. Then, the dog was returned to the starting position and E1 turned the dog away from the experimental area to prevent it from watching E2 resetting the containers.

Then, the demonstrators lined up at the starting position facing the experimental area. E2 moved the two containers to the two corners on the opposite side of the experimental area to the starting position and put treats inside them. E1 turned the dog back towards the experimental area. When the dog was calm and focused on the experimental area, E2 signaled the demonstrators to walk towards the containers. The demonstrators were split into two groups, the majority and the minority, and were instructed to walk towards different containers. The number of demonstrators in the majority group was changed in each trial. We presented the demonstrators in terms of majority and minority to all dogs at ratios of 5:1, 4:2, and 3:2 in both phases, because dogs are able to understand the quantity difference to a certain degree when objects are presented at the same time^34^. The order of these trials and the position of demonstrators were randomised.

After the demonstrators arrived at a container, they called “it looks good!” (in Japanese), whilst looking into the container. E1 kept the dog at the starting position for 3 sec after the demonstrators arrived at the container to enable it to observe the demonstrators’ behaviour, and then released the dog and allowed it to choose one of the containers (Fig. 2B). The dog obtained rewards irrespective of its choice.

In the unfamiliar phase, the demonstrators had no contact with the dog prior to and during the test session (unfamiliar demonstrators). In the familiar phase, two demonstrators interacted with and gave the dog treats for 10 min before the test session (familiar demonstrators), but had no contact with the dog during the session. In the trials, the familiar demonstrators were always assigned to the minority group. The order of the phases was counterbalanced.

### Statistical analysis

We performed a GLMM (multivariate logistic regression with mixed effects) analysis with R software^47^ by using the glmer function in the lme4 package. The objective variable was whether dogs chose the majority group, and we included dog sex, age, history (shelter/house dogs), familiarity(unfamiliar/familiar), and the ratio of demonstrators (5:1/4:2/3:2) as fixed effects, and the order of phase as a control variable. Dog’s identity was included as a random effect.

### Ethics

Ethical approval for this study was provided by the Ethics Committee of Azabu University (#131119-1), which follows “Guidelines for Proper Conduct of Animal Experiments” by the Science Council of Japan (2006).

## Supplementary information

Table S1.

## References

[1] Nagasawa M (2015). Oxytocin-gaze positive loop and the coevolution of human-dog bonds. Science.

[2] Insel TR, Young LJ (2001). The neurobiology of attachment. Nat. Rev. Neurosci..

[3] Hare B, Tomasello M (2005). Human-like social skills in dogs?. Trends Cogn. Sci..

[4] Hare B, Brown M, Williamson C, Tomasello M (2002). The domestication of social cognition in dogs. Science.

[5] Miklósi A (2003). A simple reason for a big difference: wolves do not look back at humans, but dogs do. Curr. Biol..

[6] Nagasawa M, Murai K, Mogi K, Kikusui T (2011). Dogs can discriminate human smiling faces from blank expressions. Anim. Cogn..

[7] Müller CA, Schmitt K, Barber AL, Huber L (2015). Dogs can discriminate emotional expressions of human faces. Curr. Biol..

[8] Ohkita M, Nagasawa M, Mogi K, Kikusui T (2016). Owners’ direct gazes increase dogs’ attention-getting behaviors. Behav. Processes.

[9] Duranton C, Bedossa T, Gaunet F (2019). Pet dogs exhibit social preference for people who synchronize with them: what does it tell us about the evolution of behavioral synchronization?. Anim. Cogn..

[10] Palagi E, Nicotra V, Cordoni G (2015). Rapid mimicry and emotional contagion in domestic dogs. R. Soc. Open Sci..

[11] Range F, Huber L, Heyes C (2011). Automatic imitation in dogs. Proc. Biol. Sci..

[12] Kubinyi E, Miklósi A, Topál J, Csányi V (2003). Social mimetic behaviour and social anticipation in dogs: preliminary results. Anim. Cogn..

[13] Topál J, Byrne RW, Miklósi A, Csányi V (2006). Reproducing human actions and action sequences: Do as I do! in a dog. Anim. Cogn..

[14] Kubinyi E, Topál J, Miklósi A, Csányi V (2003). Dogs (Canis familiaris) learn from their owners via observation in a manipulation task. J. Comp. Psychol..

[15] Johnston AM, Holden PC, Santos LR (2017). Exploring the evolutionary origins of overimitation: A comparison across domesticated and non-domesticated canids. Dev. Sci..

[16] Huber L, Popovová N, Riener S, Salobir K, Cimarelli G (2018). Would dogs copy irrelevant actions from their human caregiver?. Learn Behav..

[17] Claidière N, Whiten A (2012). Integrating the study of conformity and culture in humans and nonhuman animals. Psychol. Bull..

[18] Van Leeuwen EJC, Haun DBM (2014). Conformity without majority? The case for demarcating social from majority influences. Anim. Behav..

[19] Haun DBM, Rekers Y, Tomasello M (2012). Majority-biased transmission in chimpanzees and human children, but not orangutans. Curr. Biol..

[20] Haun DBM, Rekers Y, Tomasello M (2014). Children Conform to the Behavior of Peers; Other Great Apes Stick With What They Know. Psychol. Sci..

[21] Pike TW, Laland KN (2010). Conformist learning in nine-spined sticklebacks’ foraging decisions. Biol. Lett..

[22] Jolles JW, de Visser L, van den Bos R (2011). Male Wistar rats show individual differences in an animal model of conformity. Anim. Cogn..

[23] Battesti M, Moreno C, Joly D, Mery F (2012). Spread of social information and dynamics of social transmission within Drosophila groups. Curr. Biol..

[24] Van Leeuwen EJC, Haun DBM (2013). Conformity in nonhuman primates: fad or fact?. Evol. Hum. Behav..

[25] Miklósi, A. Chapter 4. A comparative approach to Canis in *Dog behaviour, evolution, and cognition*. 67–93 (Oxford University Press, 2007).

[26] Marshall-Pescini, S. & Kaminski, J. The social dog: history and evolution in *The Social* Dog*: Behavior and Cognition* (eds. Kaminski, J., Marshall-Pescini, S.) 3–33 (Academic Press, 2014).

[27] Germar M, Sultan A, Kaminski J, Mojzisch A (2018). Dogs (Canis familiaris) stick to what they have learned rather than conform to their conspecifics’ behavior. PLoS One.

[28] Fuller, T. K. Wolves: behavior, ecology and conservation in *Wolf population dynamics* (Fuller, T. K., Mech, L. D. & Cochrane, J. F. Eds.). 161–191 (University of Chicago Press, 2003).

[29] Marshall-Pescini S, Schwarz JFL, Kostelnik I, Virányi Z, Range F (2017). Importance of a species’ socioecology: Wolves outperform dogs in a conspecific cooperation task. Proc. Natl. Acad. Sci. USA.

[30] Range F, Marshall-Pescini S, Kratz C, Virányi Z (2019). Wolves lead and dogs follow, but they both cooperate with humans. Sci. Rep..

[31] Cimarelli G, Marshall-Pescini S, Range F, Virányi Z (2019). Pet dogs’ relationships vary rather individually than according to partner’s species. Sci. Rep..

[32] Daniels TJ, Bekoff M (1989). Population and social biology of free-ranging dogs, Canis familiaris. J. Mammal..

[33] West RE, Young RJ (2002). Do domestic dogs show any evidence of being able to count?. Anim. Cogn..

[34] Ward C, Smuts BB (2007). Quantity-based judgments in the domestic dog (Canis lupus familiaris). Anim. Cogn..

[35] Baker JM, Morath J, Rodzon KS, Jordan KE (2012). A shared system of representation governing quantity discrimination in canids. Front. Psychol..

[36] Kundey SM (2012). Domestic dogs’ (Canis familiaris) choices in reference to agreement among human informants on location of food. Anim. Cogn..

[37] Gácsi M, Topál J, Miklósi Á, Dóka A, Csányi V (2001). Attachment behavior of adult dogs (Canis familiaris) living at rescue centers: forming new bonds. J. Comp. Psychol..

[38] Udell MAR, Dorey NR, Wynne CDL (2008). Wolves outperform dogs in following human social cues. Animal Behav..

[39] D’Aniello B (2017). What’s the point? Golden and Labrador retrievers living in kennels do not understand human pointing gestures. Anim. Cogn..

[40] Udell MA, Dorey NR, Wynne C (2010). D.What did domestication do to dogs? A new account of dogs’ sensitivity to human actions. Biol. Rev. Camb. Philos. Soc..

[41] Katayama M (2019). Emotional contagion from humans to dogs is facilitated by duration of ownership. Front. Psychol..

[42] Marshall-Pescini S, Prato-Previde E, Valsecchi P (2011). Are dogs (Canis familiaris) misled more by their owners than by strangers in a food choice task?. Anim. Cogn..

[43] Serpell, J., Duffy, D. L. & Jagoe, J. A. Becoming a dog: Early experience and the development of behavior in *The Domestic Dog* (ed. Serpell, J.) 95–117 (Cambridge University Press, 2016).

[44] Duranton C, Gaunet F (2016). Effects of shelter housing on dogs’ sensitivity to human social cues. J. Vet. Behave..

[45] Zimen, E. *Wölfe und Königspudel: vergleichende Verhaltensbeobachtungen* (R. Piper, 1971). Translated to Japanese by Satoshi Shiraishi, 227–238 (Shisaku-sha, 1977).

[46] Marshall-Pescini S, Valsecchi P, Petak I, Accorsi PA, Previde EP (2008). Does training make you smarter? The effects of training on dogs’ performance (Canis familiaris) in a problem solving task. Behav Processes.

[47] R Core Team R: A language and environment for statistical computing. R Foundation for Statistical Computing, Vienna, Austria, https://www.R-project.org/ (2018).

