# Supplementary material for: Familiarity with humans affect dogs’ tendencies to follow human majority groups
Source: Sci Rep. 2020 Apr 28;10:7119. doi: 10.1038/s41598-020-64058-5 (PMC7188858; doi:10.1038/s41598-020-64058-5)
Supplement: Supplementary file 1 — Table S1. [file 41598_2020_64058_MOESM1_ESM.docx]

**Supplementary information**

**Familiarity with humans affect dogs’ tendencies to follow human majority groups**

Miho Nagasawa^1^, Kazutaka Mogi^1^, Hisashi Ohtsuki^2^ and Takefumi Kikusui^1^*

1 Azabu University, Department of Animal Science and Biotechnology, Sagamihara, 252-5201, Japan

2 Department of Evolutionary Studies of Biosystems, School of Advanced Sciences, SOKENDAI (The Graduate University for Advanced Studies), Hayama, 240-0193, Japan.

*Correspondence

Table S1 Information for dogs in the experiment and their choices.

| Breed | Sex | Age | History | Unfamiliar phase | | | Familiar phase | | |
| --- | --- | --- | --- | --- | --- | --- | --- | --- | --- |
|  |  |  |  | 5:1 | 4:2 | 3:2 | 5:1 | 4:2 | 3:2 |
| Mix | Intact female | 5~8 | Shelter dog | 0 | 1 | 0 | 1 | 1 | 0 |
| Bolognese | Spayed | 1 | House dog | 1 | 0 | 1 | 0 | 1 | 0 |
| Mix | Intact male | 3~5 | Shelter dog | 1 | 0 | 1 | 1 | 0 | 1 |
| Mix | Intact female | 5~8 | Shelter dog | 1 | 1 | 0 | 0 | 1 | 0 |
| Beagle | Spayed | 7 | House dog | 1 | 0 | 1 | 1 | 0 | 0 |
| Mix | Intact female | 5 | Shelter dog | 1 | 1 | 1 | 0 | 1 | 0 |
| Mix | Intact female | 5 | Shelter dog | 1 | 0 | 0 | 0 | 0 | 0 |
| Beagle | Intact male | 5 | Shelter dog | 1 | 1 | 0 | 0 | 1 | 1 |
| Mix | Intact female | 5 | Shelter dog | 1 | 1 | 0 | 1 | 0 | 1 |
| Mix | Intact female | 7 | Shelter dog | 1 | 1 | 0 | 1 | 1 | 0 |
| Bolognese | Spayed | 2 | House dog | 1 | 0 | 0 | 1 | 1 | 1 |
| Japanese Spitz | Castrated | 10 | Shelter dog | 0 | 1 | 0 | 1 | 1 | 0 |
| Mix | Castrated | 3 | Shelter dog | 1 | 0 | 1 | 1 | 1 | 0 |
| Pembroke Welsh Corgi | Castrated | 3 | Shelter dog | 1 | 1 | 0 | 1 | 0 | 0 |
| Mix | Castrated | 3 | Shelter dog | 0 | 1 | 1 | 1 | 1 | 1 |
| Mix | Castrated | 1 | Shelter dog | 1 | 1 | 0 | 0 | 1 | 0 |
| Standard Poodle | Castrated | 12 | House dog | 1 | 0 | 0 | 0 | 1 | 0 |
| Standard Poodle | Castrated | 1 | House dog | 1 | 0 | 0 | 0 | 0 | 1 |
| Standard Poodle | Intact female | 1 | House dog | 1 | 0 | 1 | 0 | 1 | 0 |
| Standard Poodle | Castrated | 1 | House dog | 1 | 0 | 1 | 1 | 0 | 1 |
| Dalmatian | Spayed | 10 | House dog | 1 | 0 | 1 | 0 | 0 | 1 |
| Dalmatian | Castrated | 8 | House dog | 1 | 0 | 1 | 1 | 0 | 0 |
| Standard Poodle | Castrated | 1 | House dog | 1 | 0 | 1 | 0 | 0 | 1 |
| Labrador retriever | Spayed | 13 | House dog | 0 | 0 | 1 | 0 | 0 | 1 |
| Standard Poodle | Spayed | 7 | House dog | 1 | 1 | 1 | 1 | 1 | 0 |
| Whippet | Spayed | 4 | House dog | 1 | 0 | 1 | 1 | 0 | 1 |
| Mix | Spayed | 3~5 | Shelter dog | 1 | 0 | 1 | 0 | 0 | 0 |
| Barneys Mountain Dog | Castrated | 5 | House dog | 0 | 1 | 0 | 0 | 1 | 1 |
| Standard Poodle | Intact female | 1 | House dog | 0 | 1 | 1 | 1 | 1 | 0 |
| Mix | Intact male | 1～2 | Shelter dog | 1 | 0 | 1 | 0 | 1 | 1 |
| Shetland Sheepdog | Castrated | 12 | House dog | 1 | 0 | 1 | 1 | 1 | 0 |
| Standard Poodle | Castrated | 6 | House dog | 0 | 0 | 1 | 1 | 0 | 1 |
| Mix | Spayed | 2 | Shelter dog | 0 | 0 | 1 | 0 | 1 | 0 |
| Miniature Dachshund | Castrated | 4 | Shelter dog | 1 | 1 | 0 | 0 | 1 | 1 |
| Mix | Castrated | 8 | Shelter dog | 0 | 1 | 0 | 0 | 0 | 0 |
| Labrador retriever | Spayed | 5 | House dog | 1 | 0 | 0 | 0 | 1 | 0 |
| Mix | Intact female | 5 | Shelter dog | 1 | 1 | 0 | 0 | 1 | 0 |
| Saluki* | Intact female | 1 | House dog | - | - | - | - | - | - |

* Excluded from analysis

“1” means that a dogs chose the majority group.
